# Teaching the musculoskeletal examination in pediatrics: a scoping review

**DOI:** 10.1186/1546-0096-10-S1-A9

**Published:** 2012-07-13

**Authors:** Clare Hutchinson, Marie-Paule Morin, Rayfel Schneider, Scott Reeves

**Affiliations:** 1 The Hospital for Sick Children, Toronto, ON, Canada; 2 Hospital for Sick Children/Sainte-Justine, Toronto, ON, Canada; 3 University of Toronto, Toronto, ON, Canada

## Purpose

To conduct a scoping review of the current evidence describing how the musculoskeletal physical examination is taught and assessed, with particular focus on the Pediatric context.

## Methods

We searched electronic databases for the past 15 years for relevant articles using any type of study design. Articles describing aspects of curricula focusing on musculoskeletal exam education were eligible for inclusion. Abstracts were reviewed, and full text articles were obtained for studies meeting inclusion criteria. Reference lists were examined and additional relevant articles were obtained. Full length articles were read, abstracted and then categorized based on which part of curricular design they describe, using Kern’s model of curriculum development for medical education as a framework to ensure that all necessary elements are considered. The six key components for curriculum design as described by Kern are: problem identification and general needs assessment; targeted needs assessment; goals and specific measurable objectives; educational strategies; implementation; evaluation, assessment and feedback.

## Results

Of 50 citations identified from the database search, 35 met inclusion criteria. An additional 33 were obtained from review of reference lists. A total of 68 papers were therefore included in this review. Using Kern’s framework it was found that the general and targeted needs assessments revealed that trainees have poor musculoskeletal knowledge, and that both teachers and students have limited confidence in their musculoskeletal assessment skills. Published goals and objectives provide lengthy lists of examination skills which do not adequately describe competent performance. Educational strategies and assessment tools to improve the musculoskeletal exam rely heavily on student self-assessment, and have not shown a clear link to increased identification of abnormalities when examining real patients. Consideration of the resources and support required for curriculum implementation, and discussions of program evaluation were generally not present. The majority of studies (n=47) focused on medical student education related to adult musculoskeletal skills, with little description of curricula dealing with the Pediatric musculoskeletal examination.

## Conclusion

The current literature leaves many gaps in our understanding of the necessary knowledge, skills and attitudes required to teach the Pediatric joint exam. Methodologically rigorous educational studies focusing on the unique aspects of conducting a musculoskeletal exam in a child are required to advance our effectiveness in teaching these skills.

## Disclosure

Clare Hutchinson: UCB, Inc., 2; Marie-Paule Morin: None; Rayfel Schneider: None; Scott Reeves: None.

